# Greater aperture counteracts effects of reduced stomatal density on water use efficiency: a case study on sugarcane and meta-analysis

**DOI:** 10.1093/jxb/erae271

**Published:** 2024-07-18

**Authors:** Daniel Lunn, Baskaran Kannan, Amandine Germon, Alistair Leverett, Tom E Clemente, Fredy Altpeter, Andrew D B Leakey

**Affiliations:** Carl R. Woese, Institute of Genomic Biology, 1206 W. Gregory Drive, University of Illinois Urbana-Champaign, Urbana, IL 61801, USA; Center for Advanced Bioenergy and Bioproducts Innovation, 1206 W. Gregory Drive, University of Illinois Urbana-Champaign, Urbana, IL 61801, USA; Department of Plant Biology, University of Illinois Urbana-Champaign, Urbana, IL 61801, USA; Center for Digital Agriculture, University of Illinois Urbana-Champaign, Urbana, IL 61801, USA; Agronomy Department, 3105 McCarty Hall B, University of Florida, Gainesville, FL 32603, USA; Center for Advanced Bioenergy and Bioproducts Innovation, 3105 McCarty Hall B, University of Florida, Gainesville, FL 32603, USA; Carl R. Woese, Institute of Genomic Biology, 1206 W. Gregory Drive, University of Illinois Urbana-Champaign, Urbana, IL 61801, USA; Center for Advanced Bioenergy and Bioproducts Innovation, 1206 W. Gregory Drive, University of Illinois Urbana-Champaign, Urbana, IL 61801, USA; Carl R. Woese, Institute of Genomic Biology, 1206 W. Gregory Drive, University of Illinois Urbana-Champaign, Urbana, IL 61801, USA; Center for Advanced Bioenergy and Bioproducts Innovation, 1206 W. Gregory Drive, University of Illinois Urbana-Champaign, Urbana, IL 61801, USA; Department of Agronomy and Horticulture, 202 Keim Hall, University of Nebraska-Lincoln, Lincoln, NE 68583, USA; Center for Advanced Bioenergy and Bioproducts Innovation, 202 Keim Hall, University of Nebraska-Lincoln, Lincoln, NE 68583, USA; Agronomy Department, 3105 McCarty Hall B, University of Florida, Gainesville, FL 32603, USA; Center for Advanced Bioenergy and Bioproducts Innovation, 3105 McCarty Hall B, University of Florida, Gainesville, FL 32603, USA; Carl R. Woese, Institute of Genomic Biology, 1206 W. Gregory Drive, University of Illinois Urbana-Champaign, Urbana, IL 61801, USA; Center for Advanced Bioenergy and Bioproducts Innovation, 1206 W. Gregory Drive, University of Illinois Urbana-Champaign, Urbana, IL 61801, USA; Department of Plant Biology, University of Illinois Urbana-Champaign, Urbana, IL 61801, USA; Center for Digital Agriculture, University of Illinois Urbana-Champaign, Urbana, IL 61801, USA; Department of Crop Sciences, University of Illinois Urbana-Champaign, Urbana, IL 61801, USA; MPI of Molecular Plant Physiology, Germany

**Keywords:** Epidermal patterning, epidermal patterning factor, stomatal conductance, stomatal density, water use efficiency

## Abstract

Stomata regulate CO_2_ and water vapor exchange between leaves and the atmosphere. Stomata are a target for engineering to improve crop intrinsic water use efficiency (iWUE). One example is by expressing genes that lower stomatal density (SD) and reduce stomatal conductance (*g*_sw_). However, the quantitative relationship between reduced SD, *g*_sw_, and the mechanisms underlying it is poorly understood. We addressed this knowledge gap using low-SD sugarcane (*Saccharum* spp. hybrid) as a case study alongside a meta-analysis of data from 10 species. Transgenic expression of EPIDERMAL PATTERNING FACTOR 2 from *Sorghum bicolor* (SbEPF2) in sugarcane reduced SD by 26–38% but did not affect *g*_sw_ compared with the wild type. Further, no changes occurred in stomatal complex size or proxies for photosynthetic capacity. Measurements of gas exchange at low CO_2_ concentrations that promote complete stomatal opening to normalize aperture size between genotypes were combined with modeling of maximum *g*_sw_ from anatomical data. These data suggest that increased stomatal aperture is the only possible explanation for maintaining *g*_sw_ when SD is reduced. Meta-analysis across C_3_ dicots, C_3_ monocots, and C_4_ monocots revealed that engineered reductions in SD are strongly correlated with lower *g*_sw_ (*r*^2^=0.60–0.98), but this response is damped relative to the change in anatomy.

## Teaser

Engineered reductions in stomatal density do not achieve equivalent reductions in stomatal conductance, probably due to compensating increases in stomatal aperture.

## Introduction

Engineering crops to have greater water use efficiency (WUE) is a key target for crop improvement to deal with the increased frequency and intensity of drought periods resulting from climate change (Leakey *et al.*, 2019). At the leaf level, intrinsic WUE (iWUE) is defined as the ratio of net photosynthetic CO_2_ assimilation (*A*_n_) relative to stomatal conductance (*g*_sw_). *A*_n_ and *g*_sw_ are generally tightly coupled (Leakey *et al.*, 2019), but if *g*_sw_ can be reduced with less or no reduction in *A*_n_, it can result in greater iWUE along with lower water use per unit of biomass production at the whole-plant scale (Yoo *et al.*, 2009). The level of *g*_sw_ is a function of stomatal density (SD), stomatal complex size, and stomatal aperture (Franks *et al.*, 2009). Although any of these parameters could be altered to lower *g*_sw_, there is a strong proof-of-concept for reducing SD by manipulating the gene network regulating stomatal development (Harrison *et al.*, 2020).

The ultimate effect of lowering SD will depend on whether changes in stomatal complex size and/or stomatal aperture coincide with amplifying or ameliorating the *g*_sw_ response. There is broad evidence that both unintended effects can co-occur with the targeted reduction in SD. For example, in Arabidopsis, pepper, poplar, and rice, engineered reductions in SD coincide with greater stomatal complex size (Zhu *et al.*, 2015; Wang *et al.*, 2016; Mohammed *et al.*, 2019; Zhao *et al.*, 2020; Li *et al.*, 2021; Karavolias *et al.*, 2023). However, the opposite response of smaller stomata in low-SD plants has been observed in barley and rice (Hughes *et al.*, 2017; Caine *et al.*, 2019). Overexpression of EPIDERMAL PATTERNING FACTOR 1 (EPF1) or knockout of EPF-Like 9 (EPFL9) increased stomatal complex size in the rice cultivar Nipponbare (Mohammed *et al.*, 2019; Karavolias *et al.*, 2023). Conversely, overexpression of EPF1 in IR64 decreased stomatal size (Caine *et al.*, 2019). In addition, engineered reductions in SD coincided with greater stomatal aperture in Arabidopsis and rice (both Nipponbare and IR64) when that was estimated from epidermal impressions/peels or inferred from increases in the ratio of operating *g*_sw_ from gas exchange to maximum *g*_sw_ from anatomical data (Büssis *et al*., 2006; Franks *et al.*, 2015; Caine *et al.*, 2019; Karavolias *et al.*, 2023). However, the conditions under which these mechanisms ameliorate or enhance the resulting impact on *g*_sw_ have not been widely tested and are poorly understood. In addition, the quantitative relationship between reduced SD and *g*_sw_ has not been broadly assessed. This study aims to address this knowledge gap by (i) reducing SD in the C_4_ species sugarcane to evaluate interactions between stomatal anatomy and leaf gas exchange fluxes; and (ii) performing a meta-analysis of the relationship between engineered reductions in SD and *g*_sw_.

C_4_ species are a potentially valuable system for engineering crops with low SD to improve iWUE. As a result of their CO_2_-concentrating mechanism and recent increases in atmospheric [CO_2_], photosynthesis in C_4_ crops is CO_2_ saturated (Leakey *et al.*, 2019; Pignon and Long, 2020). Therefore, a moderate reduction in *g*_s_ that reduces intercellular [CO_2_] (*c*_i_) to the lowest possible concentration without dropping below the inflection point of the *A*/*c*_i_ curve can lower transpiration and preserve soil moisture to avoid drought stress without increasing stomatal limitation to *A*_n_. The evidence for this comes from (i) leaf-level gas exchange modeling and process-based crop modeling (Leakey *et al.*, 2019; Pignon and Long, 2020); (ii) FACE (free air CO_2_ enrichment) experiments where photosynthesis or biomass production was not stimulated when C_4_ crops are grown at elevated [CO_2_] and with adequate water supply (Leakey *et al.*, 2009; Markelz *et al.*, 2011; Kellner *et al.*, 2019); and (iii) transgenic sorghum and maize with low SD (Liu *et al.*, 2015; Ferguson *et al.*, 2024). In this study, C_4_ species are an attractive system because it should be possible to study interactions between *g*_sw_, SD, and stomatal complex size as they vary without changes in photosynthetic capacity or *A*_n_. In contrast, photosynthesis in C_3_ species under typical growth conditions is sensitive to variations in intracellular CO_2_. Thus, reducing *g*_sw_ in C_3_ species lowers *c*_i_, resulting in increased stomatal limitation of *A*_n_ (Farquhar and Sharkey, 1982). Most studies of low-SD C_3_ plants show this phenomenon, with *A*_n_ values lower than the wild type (Hughes *et al.*, 2017; Caine *et al.*, 2019; Dunn *et al.*, 2019; Li *et al.*, 2021; Karavolias *et al.*, 2023). Therefore, since *g*_sw_ is also tightly coupled to *A*_n_, any potential feedback between the two complicates the interpretation of structure–function relationships.

Some of the mechanisms that may act to modify the effect on *g*_sw_ from reducing SD are developmental. Overexpression of EPFs is understood to suppress SD via a reduction in stomatal index that results from cells halting progression at multiple stages of stomatal development (Hughes *et al.*, 2017; Caine *et al.*, 2019). However, less is understood about the downstream aspects of engineering low-SD plants on other aspects of epidermal patterning that could impact leaf gas exchange. A recent report showed that increasing SD did not impact bulliform density but increased hair and silica cells (Abrash *et al.*, 2018). These structures should not be overlooked since hair cell types play an important role in boundary layer conductance that can impact gas exchange (Bickford, 2016). Given the significance of stomatal clustering to gas exchange (Dow *et al.*, 2014), important differences could result from whether reduced SD arises due to fewer stomata per cell file and/or fewer files of cells containing stomata on a grass leaf. Signals that impact stomatal development can also modify other epidermal cell types, such as hair cells (Torii, 2021). Alterations in the proportion and development of different cell types may provide feedback on how stomatal complex size is determined. However, despite discovering some genes that regulate stomatal complex size, this process is less well understood than cell fate regulation (Des Marais *et al.*, 2014; Nunes *et al.*, 2020).

The network of genes regulating stomatal development is a model system for studying the regulation of cell fate and has been extensively elucidated in Arabidopsis (Simmons and Bergmann, 2016). The collective function of these genes is largely conserved between C_3_ dicots and grasses, but some specifics of individual gene function have diverged (McKown and Bergmann, 2020). The potential for novel function is highlighted by the dumbbell-shaped guard cells flanked by a subsidiary cell that distinguishes grass stomata from the classic kidney-shaped guard cells of dicots (Nunes *et al.*, 2020). Establishing guard cell identity in *Brachypodium* and rice requires transcription factors INDUCER OF CBP EXPRESSION 1 (ICE1) and one active copy of either SPEECHLESS 1 (SPCH1) or SPEECHLESS 2 (SPCH2) (Raissig *et al.*, 2016; Wu *et al.*, 2019). The resulting SPCH:ICE heterodimer stimulates gene expression whereby precursor cells asymmetrically divide to produce a guard mother cell and a pavement cell. Lateral signals then radiate from the guard mother cell to stimulate subsidiary cell formation before a final longitudinal division produces mature guard cells (Lai *et al.*, 2005; Raissig *et al.*, 2017). Stomatal spacing is partly regulated via signaling peptides from the EPF family. Upon initiation of stomatal fate, the SPCH:ICE complex stimulates the production of EPFs in the guard mother cell. In Arabidopsis, these EPF peptides radiate from the guard mother cell and compete with the positive regulator STOMAGEN (Sugano *et al.*, 2010) to bind ERECTA/ERECTA-LIKE (ER/ERL) receptors in a concentration-dependent manner (Torii, 2021). The binding triggers a mitogen-activated protein kinase signaling cascade that passes through the EMBRYO DEFECTIVE 71 (YDA) gene to phosphorylate SPCH (Bergmann *et al.*, 2004). Phosphorylation of SPCH inhibits SPCH:ICE heterodimerization that prevents stomatal fate in the surrounding pavement cells (Abrash *et al.*, 2018).

Expressing or overexpressing EPF family members has resulted in reduced SD in many C_3_ species, suggesting that function is largely conserved across C_3_ monocots and dicots (Hepworth *et al.*, 2015; Wang *et al.*, 2016; Hughes *et al.*, 2017; Caine *et al.*, 2019; Dunn *et al.*, 2019; Mohammed *et al.*, 2019; Jiao *et al.*, 2022; Karavolias *et al.*, 2023). However, the possibility of neofunctionalization or functional redundancy among EPFs in C_4_ species has received less attention despite evidence that the evolution of Kranz anatomy is associated with changes in the role of SHR and SCARECROW in leaf development (Schuler *et al.*, 2018; Hughes *et al.*, 2023). In sorghum, overexpression of EPF1 had very mild effects on SD relative to rice (Caine *et al.*, 2019), wheat (Dunn *et al.*, 2019), or barley (Hughes *et al.*, 2017), while expression of a synthetic EPF2 reduced SD and increased iWUE similar to other species (Franks *et al.*, 2015; Ferguson *et al.*, 2024). Therefore, the apparent functional conservation of EPF genes means that they are excellent candidates for producing model systems to study the effects of reduced SD in C_4_ species.

Our study was motivated by the need to understand better the relationship between SD and *g*_sw_ in crop species. Sugarcane was explored as a case study system for C_4_ species. Sugarcane is the leading crop in biomass production and provides 80% of the global table sugar and 40% of the world’s biofuel (Budeguer *et al.*, 2021). We aimed to (i) modulate an EPF gene to engineer sugarcane with lower SD and *g*_sw_; (ii) quantify changes in epidermal cell patterning and stomatal complex size; (iii) estimate the consequences of altered stomatal patterning for maximum potential *g*_sw_; (iv) assess changes in *g*_sw_ and photosynthetic physiology arising from altered SD; and (v) perform a meta-analysis to summarize the quantitative relationship between lower SD and reduced *g*_sw_ across C_3_ and C_4_ species from dicot and monocot lineages.

## Materials and methods

### Construct design and generation

A BLAST search of the *Sorghum bicolor* genome using *Arabidopsis thaliana* (Arabidopsis) EPF2 (At1G34254) identified Sobic.006G104400 with 69% sequence identity that we named SbEPF2. We amplified this sequence from sorghum before cloning the amplicon into plasmid pPTN1434 to create expression cassettes containing SbEPF2 and neomycin phosphotransferase II (*npt*II). The 5'-untranslated region (UTR), EPF2 coding sequence, and 3'-UTR from *S. bicolor* were used for this vector construct. SbEPF2 was placed under transcriptional control of the ubiquitin promoter from *Zea mays* with its first intron (ZmUbi) and the 35S poly(A) signal from cauliflower mosaic virus (CaMV). The selectable marker gene *npt*II was under transcriptional control of the 35S promoter and the 35S poly(A) signal from the CaMV.

### Generation of transgenic sugarcane

Plasmid pPTN1434, containing SbEPF2 and *npt*II expression cassettes, was introduced into sugarcane callus CP88-1762 by biolistic gene transfer as described by Taparia *et al.* (2012). Briefly, leaf whorl cross-sections of CP88-1762 were cultured on modified Murashige and Skoog medium (MS) with B5 vitamins (PhytoTech Labs, KS, USA) supplemented with 3 mg l^–1^ 2,4-dichlorophenoxyacetic acid (PhytoTech Labs, KS, USA) to initiate embryogenic calli. Leaf whorl cultures were maintained at 28 °C in an incubator in the dark and subcultured every 14 d. Eight weeks after culture initiation, linearized expression cassettes were precipitated onto 1 µm gold particles and introduced into embryogenic calli using a biolistic PDS-1000/He delivery system (Bio-Rad, Hercules, CA, USA). One week after gene transfer, calli were transferred to a medium containing 20 mg l^–1^ geneticin (PhytoTech Labs) to select transgenic events. Following 5 weeks of antibiotic selection, calli were subcultured onto regeneration medium [MS medium with B5 vitamins, supplemented with 1.86 mg l^–1^ α-naphthaleneacetic acid (NAA; PhytoTech Labs] and 0.09 mg l^–1^ 6-benzylaminopurine (BAP; PhytoTech Labs), and were maintained at 28 °C in an incubator with a 16/8 h light (100 µmol m^–2^ s^–1^) and dark cycle. Regenerated shoots >1 cm in length were transferred to a modified MS basal medium with 4.4 g l^–1^ Gamborg vitamins (PhytoTech Labs) for shoot elongation and rooting. Rooted plantlets were transferred to soil and acclimatized in a temperature-controlled plant growth chamber maintained at 28 °C day and 22 °C night temperature with a 16/8 h light (400 µmol m^–2^ s^–1^) and dark cycle for further analysis.

### Analysis of transgene expression by quantitative reverse transcription–PCR

Transcript levels of SbEPF2 in the wild type and transgenics were determined by quantitative reverse transcription–PCR (RT–qPCR) analysis. We sampled the differentiation zone above the ligule from the wild type, plus events 15 and 38. We extracted RNA from these samples using the Monarch Total RNA Extraction and Purification kit (New England Biolabs). The RNA was normalized between samples to 100 ng µl^–1^ before conducting the RT–qPCR using the Luna One-Step RT-qPCR kit (New England Biolabs) on a BioRad CFX Connect Real-Time System (BioRad). Transcript levels of SbEPF2 were normalized against the glyceraldehyde-3-phosphate dehydrogenase (ShGAPDH) gene. Primers used were ShGAPDH_forward (CACGGCCACTGGAAGC), ShGAPDH_reverse (TCCTCAGGGTTCCTGATGCC), SbEPF2_forward (CGACGAGCTAGCAGGAAGAG), and SbEPF2_reverse (GGGGATCCTGTGATGTGAGC).

### Experimental design and environmental conditions for phenotyping assays

The experimental materials were vegetatively propagated, transgenic events from sugarcane cultivar CP 88-1762. Following regeneration of putative transgenic plants, PCR was used as the initial screen to confirm transgene presence in genomic DNA extracts. One plant from each of the 18 independent events was screened for reduced SD before selecting the two with the highest decrease (SbEPF2-15 and SbEPF2-38) for further propagation and testing. Ten vegetatively propagated replicates of each transgenic event plus the wild type were grown in a randomized block design for detailed phenotyping. Stomatal density, leaf photosynthetic gas exchange, specific leaf area (SLA), leaf N content, and chlorophyll content were assessed on all plants. Additional detailed evaluation of epidermal patterning and anatomical determinants of maximum *g*_s_ (*g*_smax_) were assessed on SbEPF2-15. All plants were grown under greenhouse conditions where a 15 h photoperiod was provided by combining natural irradiance with supplemental LEDs with a minimum light intensity threshold of 1500 µmol m^–2^ s^–1^. Air temperature was controlled between 27 °C and 30 °C, and 21 °C and 23 °C during day and night periods, respectively.

### Measuring stomatal density and spatial patterning

An optical tomographer was used to rapidly image the leaf epidermis using established protocols (Xie *et al.*, 2021). Each genotype was sampled by cutting a small section from a central position on the youngest fully expanded leaf. Samples were placed on dry ice and imaged within 30 min. A MarSurf CM Explorer (Mahr) scanned the leaf sample at ×20 magnification to produce images with a size of 800×800 µm^2^. The upper and lower *z*-scale limits were set manually to capture all stomata in four fields of view interspersed along a transect from the midrib to the margin of each leaf surface. The images were measured for traits relating to stomata density, stomatal epidermal patterning, bulliform epidermal patterning, prickle epidermal patterning, and stomatal complex dimensions. Average values from the four fields of view formed the technical replicate of each biological replicate. Cell counts, cell sizes, and spatial measurements were taken with ImageJ Fiji using the schema shown in the appropriate figures. Calculations of maximum aperture size and maximum theoretical stomatal conductance (*g*_smax_) were made as detailed in Al-Salman *et al.* (2023). Maximum pore aperture (*a*_max_) was calculated in µm^2^ by multiplying stomatal complex width (Sto_width_) with length (Sto_length_).


$$\begin{array}{l} a_{max}=Sto_{width}\times Sto_{length} \end{array}$$

*g*_smax_ was calculated in mol m^–2^ s^–1^. Here *d*=diffusivity of water vapor in air; *v*=molar air volume of 24.8 mol^–1^ dm^3^ under reference atmospheric pressure and greenhouse temperature of 30 °C; *I*=stomatal pore depth assumed to be Sto_width_/2; SD=stomatal density; and *a*_max_=maximum pore aperture.


$$\begin{array}{l} g_{s\max}=\frac{d}{v}\left(\frac{SD\times a_{\max}}{I+\frac{\pi }{2}\sqrt{\frac{a_{\max}}{\pi }}}\right) \end{array}$$

### Gas exchange measurements

Steady-state, light-saturated gas exchange measurements were taken using the LI-COR 6800 gas analyzer (LI-COR Biosciences, Lincoln, NE, USA). All measurements were made in the center of the youngest fully expanded leaf. Chamber conditions were maintained at a photosynthetic photon flux density (PPFD) of 2000 µmol m^–2^ s^–1^ (10% blue and 90% red light), chamber [CO_2_] of 450 ppm, temperature of 30 °C, and 60–70% relative humidity to assess leaf gas exchange at growth [CO_2_]. Additional data were collected after making step-changes in [CO_2_] from 450 to 250, 50, and 20 ppm once equilibrium was reached in the chamber to assess leaf gas exchange under conditions that promote stomatal opening.

### Leaf chlorophyll, nitrogen content, and specific leaf area

Relative chlorophyll content was estimated using a handheld SPAD-502 Plus chlorophyll meter (Konica Minolta, Tokyo, Japan). Leaf N content and SLA were assessed following the protocols of Markelz *et al.* (2011). Leaves were sampled in the greenhouse using a hole punch with an area of 4 cm^2^. These leaf discs were transferred to a drying oven set at 60 °C for 2 weeks. The dry mass of each sample was determined before being combusted in an elemental analyzer (Elemental Combustion System, Costech). The SLA was calculated as the ratio of leaf area to dry leaf mass (cm^2^ g^–1^).

### Meta-analysis

The literature was screened for experiments where *g*_sw_ was measured in transgenic plants with low SD as a result of perturbing expression of genes activating the SPCH/ICE basic helix–loop–helix (bHLH) stomatal development pathway. The search terms used in our searches were ‘stomatal density’ or ‘stomatal conductance’ with ‘WUE’. Each hit in the literature search was used to mine other studies using their reference section. This approach produced a dataset for 10 species from three functional groups, comprising *A. thaliana* (C_3_ dicot), *Hordeum vulgare* (barley, C_3_ monocot), *Oryza sativa* (rice, C_3_ monocot), *Populus tremuloides* (poplar, C_3_ dicot), *Saccharum* spp. (sugarcane, C_4_ monocot), *Solanum lycopersicum* (tomato, C_3_ dicot), *Sorghum bicolor* (sorghum, C_4_ monocot), *Triticum aestivum* (wheat, C_3_ monocot), *Vitis vinifera* (grape, C_3_ dicot), and *Zea mays* (maize, C_4_ monocot), along with the data reported in the current study (data sources: Dow *et al.*, 2014; Hepworth *et al.*, 2015; Liu *et al.*, 2015; Shen *et al.*, 2015; Wang *et al.*, 2016; Hughes *et al.*, 2017; Caine *et al.*, 2019; Dunn *et al.*, 2019; Mohammed *et al.*, 2019; Li *et al.*, 2021; Clemens *et al.*, 2022; Jiao *et al.*, 2022; Karavolias *et al.*, 2023). Data were gathered from multiple transgenic events in a single study if SD and g_sw_ were reported for each line. Studies in the meta-analysis did not discriminate between plants in growth chambers and greenhouse conditions. We calculated the percentage changes in SD and *g*_sw_ relative to the wild type to normalize the data between studies. The data were limited to measurements of *g*_s_ collected on leaves experiencing a CO_2_ range between 400 ppm and 500 ppm to simulate ambient atmospheric conditions.

### Statistical analysis and modeling

Plants were independent replicates of each genotype with *n*=10. The initial screen was analyzed using a two-way ANOVA, with all subsequent analyses conducted using a linear mixed-effects model with post-hoc pairwise comparisons. The model fitted a fixed dependent and independent variable, with the random effect being block number using the Python library Pymer4 (Jolly, 2018). Meta-analysis was conducted by linear regression using a least-squares method with the linegress function in the Python library SciPy.

## Results

**Fig. 1.**
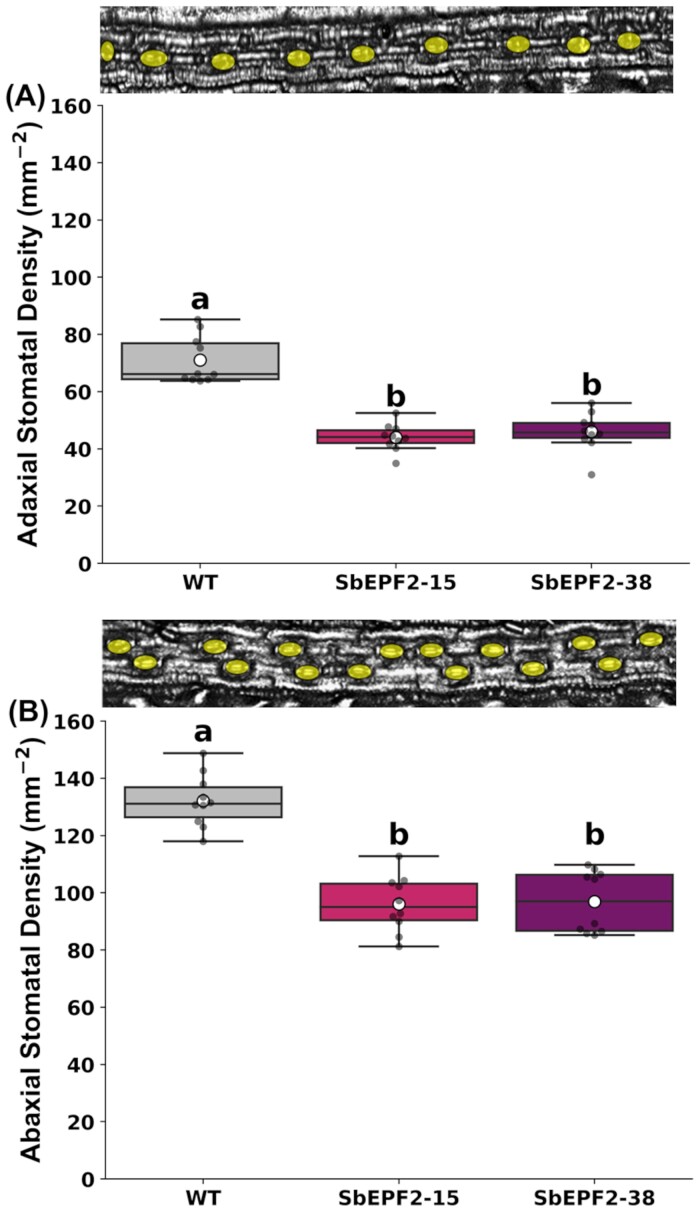
Stomatal density of wild-type (WT) transgenic sugarcane overexpressing SbEPF2 (SbEPF2-15 and SbEPF2-38). (A) Adaxial and (B) abaxial stomatal density (mm^–2^), with images above providing an example of WT stomatal patterning. Graphs show box plots of the 25th and 75th percentile, with a line indicating the median value. Each box plot shows the mean with a white circle, and gray dots showing individual observed residual data points (*n*=10), where letters within each panel indicate significant differences between genotypes.

We hypothesized that overexpressing an EPF2 ortholog in sugarcane would increase the negative regulation of stomatal development and reduce SD. On average, across 18 SbEPF2-expressing transgenic events in an initial screen, SD was 17% lower than in the wild type on the abaxial leaf surface (Supplementary Fig. S1). We selected two lines (SbEPF2-15 and SbEPF2-38) where the reduction in SD compared with the wild type was stronger than average. After vegetatively propagating lines SbEPF2-15 and SbEPF2-38 to the following generation, we sampled the stomatal development zone in leaves to confirm the presence of SbEPF2 by RT–qPCR. Transcriptional analysis showed that both transgenic lines had significant SbEPF2 expression in the leaf stomatal development zone, but no signal was detected in wild-type plants (Supplementary Fig. S2). The more replicated assessment of SD showed that each transgenic line displayed a stronger reduction in SD. Adaxial SD was 38% and 35% lower than in the wild type in SbEPF2-15 and SbEPF2-38, respectively (Fig. 1A). Abaxial responses were weaker, with SD being 26% lower than that of the wild type in both transgenic lines (Fig. 1B).

**Fig. 2.**
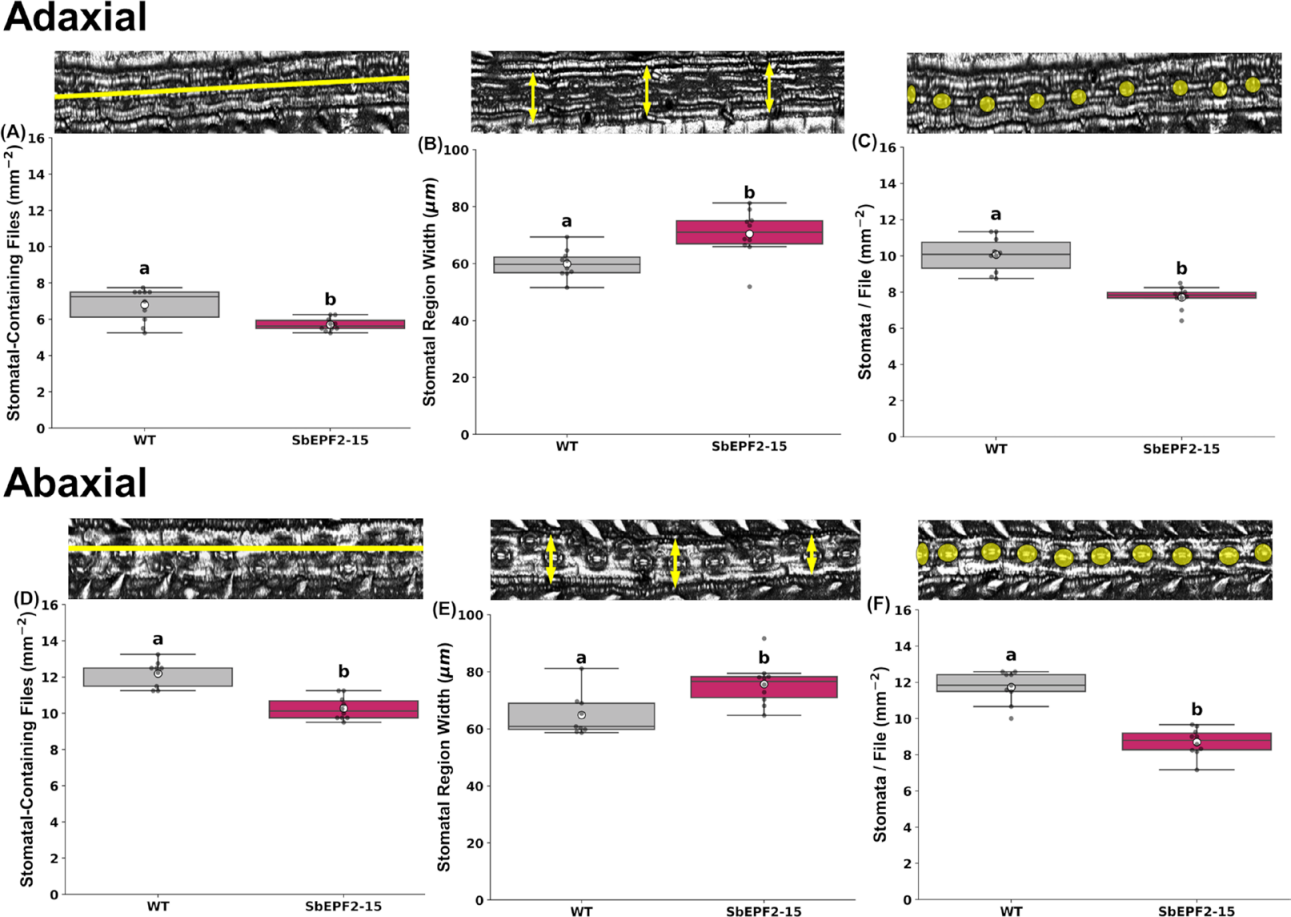
Stomatal epidermal patterning of wild-type (WT) transgenic sugarcane overexpressing SbEPF2 (SbEPF2-15). (A) Adaxial stomata-containing files; (B) adaxial stomatal region width (µm); (C) adaxial stomata per file; (D) abaxial stomata-containing files; (E) abaxial stomatal region width (µm); and (F) abaxial stomata per file with WT images above showing how each trait was measured. Graphs show box plots of the 25th and 75th percentile, with a line indicating the median value. Each box plot shows the mean with a white circle, and gray dots showing individual observed residual data points (*n*=10), where letters within each panel indicate significant differences between genotypes.

Sugarcane epidermal cells are arranged in straight files that run in a longitudinal direction along the leaf (Fig. 2). Additional detailed evaluation of epidermal patterning in SbEPF2-15 showed that the reduction in SD on both leaf surfaces compared with the wild type was driven by a reduction in the: (i) density of cell files containing stomata (adaxial –21%, abaxial –9%, Fig. 2A, D) and (ii) density of stomata within cell files (adaxial –24%, abaxial –19%; Fig. 2C, F); that is, greater spacing between stomata. The greater spacing between stomatal cell files was itself partly due to ‘stomatal regions’ (i.e. zones of intercostal cells, not including bulliform cells) being wider (adaxial +17%, abaxial +17%) in SbEPF2-15 than the wild type (Fig. 2B). Almost all stomatal regions on the adaxial surface contained only a single file of stomata surrounded by intercostal pavement cells and this did not differ between SbEPF2-15 and the wild type (Supplementary Fig. S3A). By contrast, 44% of stomatal regions on the abaxial surface contained multiple files of stomata (Supplementary Fig. S3B). The increased number of stomatal regions with multiple stomata-containing files contributed to the greater SD on the abaxial versus adaxial surface but did not differ between SbEPF2-15 and the wild type.

**Fig. 3.**
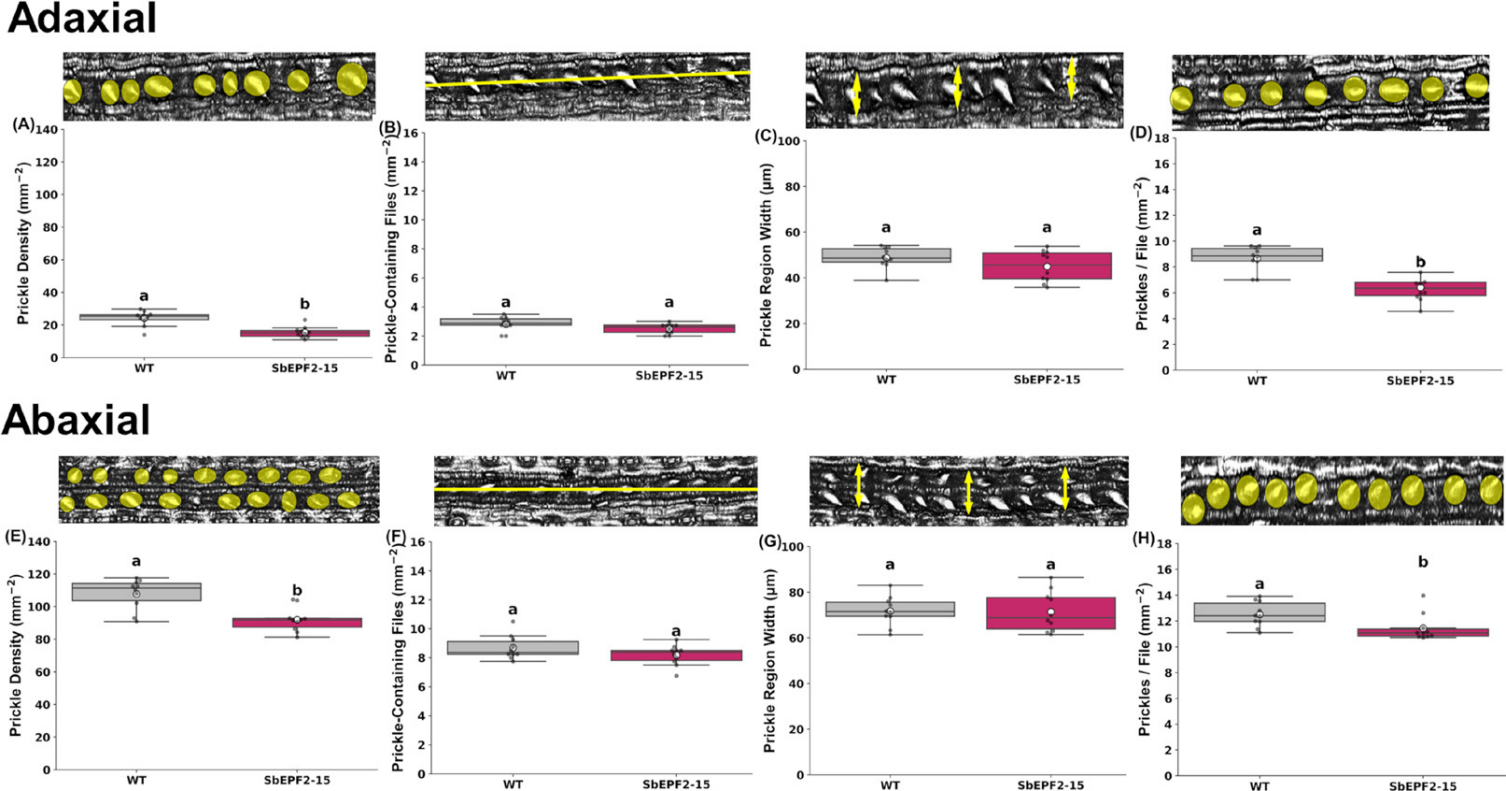
Prickle epidermal patterning of wild-type (WT) transgenic sugarcane overexpressing SbEPF2 (SbEPF2-15). (A) Adaxial prickle density (mm^–2^); (B) adaxial prickle-containing files; (C) adaxial prickle region width (µm); (D) adaxial prickles per file; (E) abaxial prickle density (mm^–2^); (F) abaxial prickle-containing files; (G) abaxial prickle region width (µm); and (H) abaxial prickles per file, with WT images above showing how each trait was measured. Graphs show box plots of the 25th and 75th percentile, with a line indicating the median value. Each box plot shows the mean with a white circle, and gray dots showing individual observed residual data points (*n*=10), where letters within each panel indicate significant differences between genotypes.

We measured the same traits in prickle and bulliform cells to study the impact of reduced SD on other major epidermal cell types. SbEPF2-15 was not significantly different from the wild type in the density of bulliform cells, the density of bulliform cell files, the width of bulliform regions, or the frequency of bulliform cells within a file (Supplementary Fig. S4). By contrast, the prickle density of SbEPF2-15 was lower than that of the wild type by 35% on the adaxial surface and 14% on the abaxial surface (Fig. 3A, E). Unlike stomata, the reduction in prickle density was not associated with a significant change in the density of cell files containing prickles (Fig. 3B, F) and was dominated by a reduction in the density of prickles within a cell file (adaxial –25%, abaxial 8%; Fig. 3D, H). Correspondingly, there was no difference between SbEPF2-15 and the wild type in the width of prickle regions (i.e. costal zones; Fig. 3C, G; Supplementary Fig. S5).

**Fig. 4.**
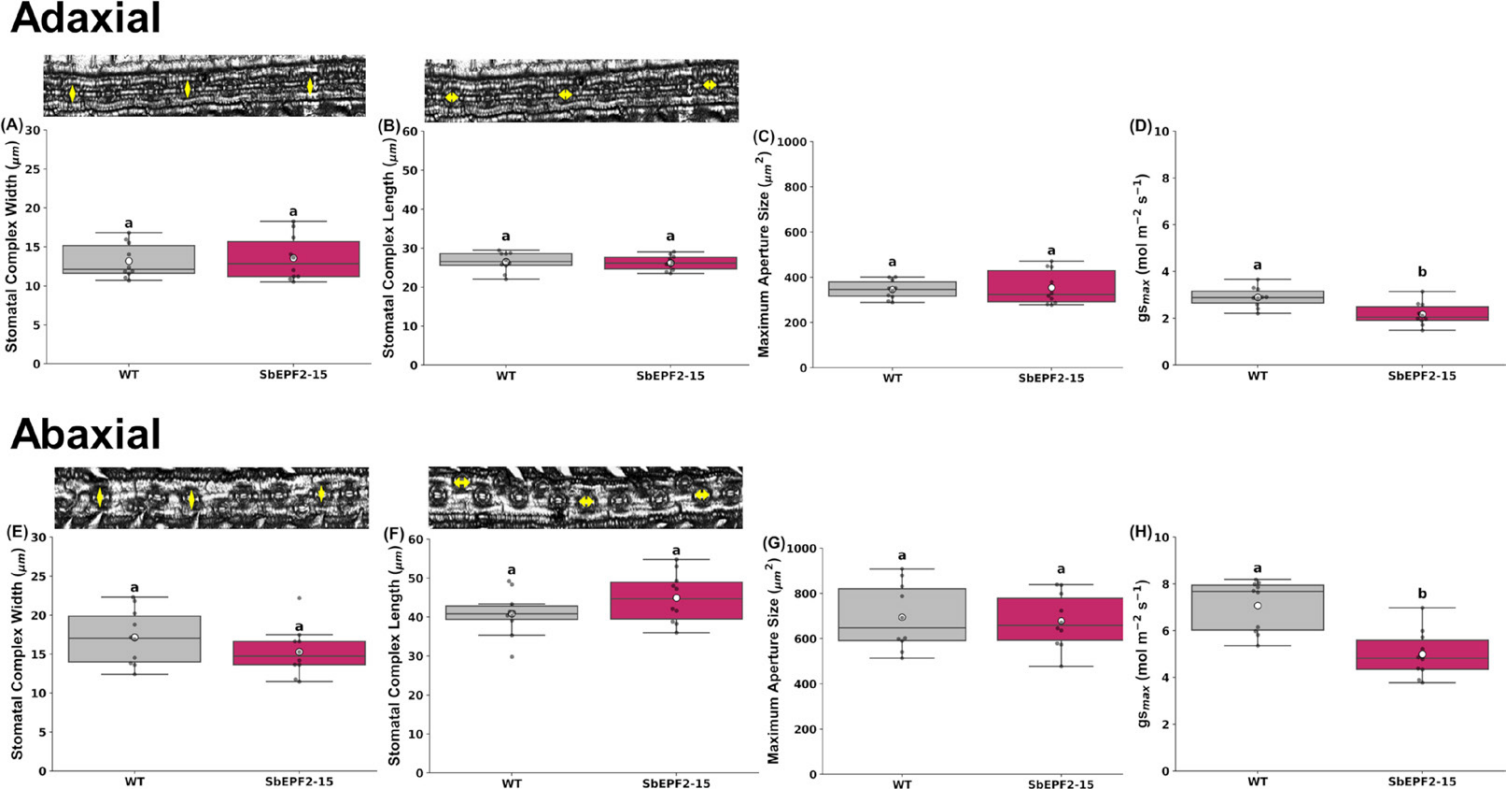
Stomatal complex measurements of wild-type (WT) transgenic sugarcane overexpressing SbEPF2 (SbEPF2-15). (A) Adaxial stomatal complex width (µm); (B) adaxial stomatal complex length (µm); (C) adaxial *A*_max_ (µm^2^); (D) adaxial *g*_smax_; (E) abaxial stomatal complex width (µm); (F) abaxial stomatal complex length (µm); (G) abaxial *A*_max_ (µm^2^); and (H) abaxial *g*_smax_, with WT images above showing how each trait was measured. Graphs show box plots of the 25th and 75th percentile, with a line indicating the median value. Each box plot shows the mean with a white circle, and gray dots showing individual observed residual data points (*n*=10), where letters within each panel indicate significant differences between genotypes.

We measured stomatal complex size and then calculated maximum aperture size and *g*_smax_ to determine if reduced SD altered these traits. Reduced SD in SbEPF2-15 compared with the wild type was not accompanied by significant changes in stomatal complex length or width on either leaf surface (Fig. 4A, B, E, F). Consequently, there was no significant difference between SbEPF2-15 and the wild type in the modeled maximum stomatal aperture on either leaf surface (Fig. 4C, G). However, the reductions in SD led to significantly lower *g*_smax_ in SbEPF2-15 compared with the wild type (–24% adaxial, –30% abaxial; Fig. 4D, H).

**Fig. 5.**
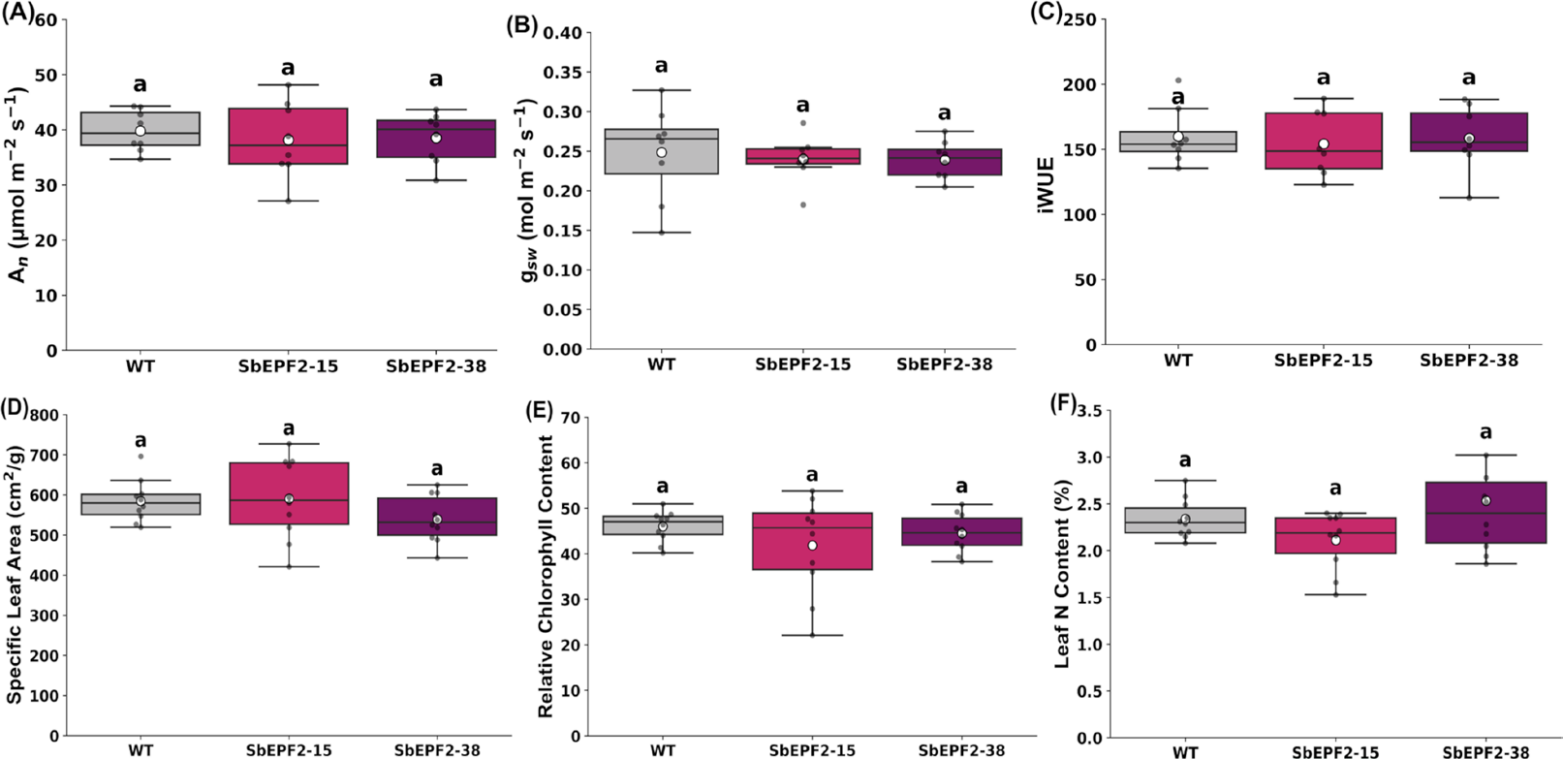
Leaf physiology of wild-type (WT) transgenic sugarcane overexpressing SbEPF2 (SbEPF2-15 and SbEPF2-38). (A) Carbon assimilation (*A*_n_); (B) stomatal conductance (*g*_sw_); (C) intrinsic water use efficiency (iWUE); (D) specific leaf area (cm^2^ g^–1^); (E) relative chlorophyll content (SPAD values); and (F) leaf nitrogen content (%). Graphs show box plots of the 25th and 75th percentile, with a line indicating the median value. Each box plot shows the mean with a white circle, and gray dots showing individual observed residual data points (*n*=10), where letters within each panel indicate significant differences between genotypes.

**Fig. 6.**
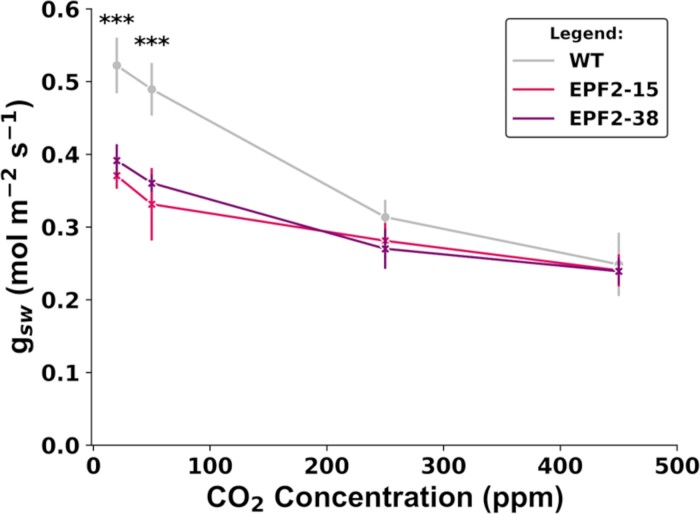
Stomatal conductance of wild-type (WT) transgenic sugarcane overexpressing SbEPF2 (SbEPF2-15 and SbEPF2-38) at varying CO_2_ concentrations. Error bars show the SE (*n*=10), with asterisks above each point indicating significant differences genotypes.

We measured gas exchange and proxies for photosynthetic capacity in the two low-SD transgenic lines. There were no significant differences between either transgenic or wild type in *A*_n_ (Fig. 5A), *g*_sw_ (Fig. 5B), or iWUE (Fig. 5C). There were also no significant differences between the wild type and either transgenic line in SLA (Fig. 5D), relative chlorophyll content (Fig. 5E), or leaf N concentration (Fig. 5F). We then re-measured *g*_sw_ at a range of [CO_2_] to explore how stomatal aperture may be modulating the effects of SD on leaf gas exchange (Fig. 6). Consistent with our previous results, there was no difference in *g*_sw_ between either transgenic line and the wild type at 450 ppm. However, as [CO_2_] was progressively reduced, the transgenic lines did not increase *g*_sw_ in the same manner as the wild type. Consequently, when stomata were open close to their maximum extent at 20 ppm CO_2_, *g*_sw_ of the transgenic lines was reduced relative to the wild type by 24–28% (Fig. 6).

**Fig. 7.**
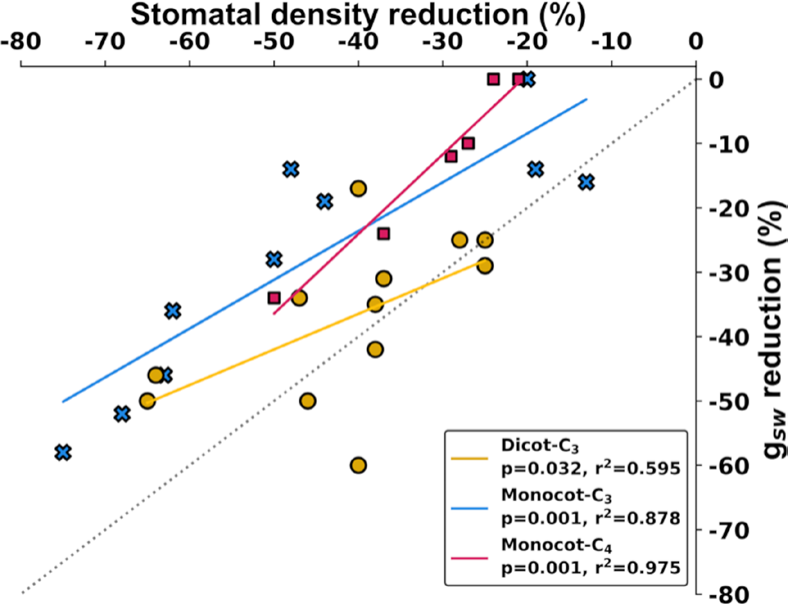
Meta-analysis of the relationship between reduced stomatal density and lower stomatal conductance in transgenic species. The data show the SD and *g*_sw_ for individual transgenic events from publications listed in the Materials and methods. Shapes show C_3_ dicot (yellow circles), C_3_ monocot (blue crosses), and C_4_ monocot (red squares) species. Trend lines were calculated using linear regression with the least-squares method. Lines show the 1–1 intercept (gray) and linear regression for C_3_ dicots (yellow), C_3_ monocots (blue), and C_4_ monocots (red). The linear regression equations are for C_3_ dicots: *y*=14.36 + 0.553*x*; C_3_ monocots *y*= –6.694 + 0.757*x*; and C_4_ monocots *y*= –25.427 + 1.237*x*.

The gas exchange measurements made us question the relationship between SD and *g*_sw_ in plants engineered to have reduced SD. Meta-analysis of previously published data revealed a significant correlation between engineered reductions in SD and reductions in *g*_sw_ (Fig. 7), with the relationship being strong for C_4_ monocots (*P=*0.001, *r*^2^=0.97) and C_3_ monocots (*P=*0.001, *r*^2^=0.87), but modest for C_3_ dicots (*P=*0.032, *r*^2^=0.59). In most cases, the reduction in *g*_sw_ was smaller than the reduction in SD. It was especially clear in monocots, where the reduction in *g*_sw_ only matched the magnitude of the reduction in SD in one case, and the intercept of the regression lines indicated that no change in *g*_sw_ would be expected given a 7–25% change in SD for C_3_ and C_4_ species, respectively.

## Discussion

This study advanced understanding of how altering the expression of stomatal development genes influences epidermal anatomy and leaf photosynthetic gas exchange by successfully addressing five aims. First, sugarcane, a species representing understudied but agriculturally important polyploid C_4_ grass crops, was engineered to have reduced SD. Second, reductions in SD were found to result in roughly equal reductions in the density of cell files containing stomata and decreases of stomata within a cell file. The altered epidermal pattern was accompanied by reductions in the density of prickle cells but not bulliform cells. Reducing SD did not alter the stomatal complex size. Third, reducing SD by 38% on the adaxial surface and 26% on the abaxial surface was predicted to reduce *g*_smax_ by 24% on the adaxial surface and 30% on the abaxial surface from our meta-analysis (Fig. 7). Fourth, there was no commensurate change in operating *g*_sw_ when it was assessed by measurement of leaf gas exchange, along with no change in *A*_n_ or proxies for photosynthetic capacity (leaf N, SLA, and chlorophyll content). This outcome appears to have been driven by increased stomatal aperture that completely compensated for the reduction in SD. Fifth, meta-analysis revealed that ameliorating the effects of reduced SD on *g*_sw_ by increasing stomatal complex size and/or stomatal aperture is likely to be a general phenomenon across C_3_ and C_4_ species from both monocot and dicot lineages. The sugarcane engineered in this study would represent the most extreme case reported to date of stomatal behavior ameliorating the effects of stomatal anatomy on *g*_sw_. It has the potential to provide a powerful experimental system for future studies on the mechanisms underlying a phenomenon with important implications for efforts to improve crop WUE.

Reducing stomatal conductance by lowering SD has proven successful in engineering crops with increased iWUE (Dow *et al.*, 2014; Hepworth *et al.*, 2015; Liu *et al.*, 2015; Shen *et al.*, 2015; Wang *et al.*, 2016; Caine *et al.*, 2019; Dunn *et al.*, 2019; Mohammed *et al.*, 2019; Li *et al.*, 2021; Clemens *et al.*, 2022; Jiao *et al.*, 2022; Karavolias *et al.*, 2023). However, C_4_ species have been understudied in this regard despite the potential for beneficial outcomes (Leakey *et al.* 2019). In addition, the quantitative relationship between engineered changes in SD and *g*_sw_ has not been summarized despite evidence for compensatory changes in stomatal complex size and stomatal aperture. To further explore this relationship in a C_4_ species, we reduced SD in sugarcane by expressing a sorghum EPF. We identified a potential EPF ortholog Sobic.006G104400 in sorghum (SbEPF2) that was 69% identical to Arabidopsis AtEPF2 (Hunt and Gray, 2009). When expressed in sugarcane, SbEPF2 decreased adaxial and abaxial SD by 35–38% and 26%, respectively (Fig. 2). These *in silico* and phenotypic data show SbEPF2 encodes a functional EPF ortholog. Qualitatively, functional conservation of SbEPF2 in sugarcane was expected, given that other experiments reported reduced SD when overexpressing EPFs in various species (Hughes *et al.*, 2017; Caine *et al.*, 2019; Dunn *et al.*, 2019; Mohammed *et al.*, 2019; Karavolias *et al.*, 2023). However, the reduction in SD was lower in our transgenics than those observed when a native EPF was overexpressed in grass species. Interestingly, transgenically expressing EPFs from various grass species in Arabidopsis produced a far lower reduction of SD than when overexpressed in the native species in several other reports (Hughes *et al.*, 2017; Caine *et al.*, 2019; Dunn *et al.*, 2019). The relatively low SD reduction observed in this report and other expression experiments may be due to sorghum and sugarcane EPF2 orthologs not having complete functional conservation. Sugarcane expressing SbEPF2 may inefficiently bind ER/ERL receptors, limiting the strength of negative repression relative to native EPF orthologs. How efficiently non-native EPF genes bind ER/ERL compared with the native EPF peptides is unknown and deserves future exploration.

Previous experiments that utilize EPFs in grasses to lower SD reported that this was driven by reducing the stomatal index (i.e., ratio of stomata to number of epidermal cells) as a result of stomatal development arresting at several different stages (Hughes *et al.*, 2017; Caine *et al.*, 2019; Dunn *et al.*, 2019). In grasses, variation in SD can also be characterized by changes in the number of cell files containing stomata and the density of stomata within a cell file. In sugarcane, both of these mechanisms contributed to the reduction in SD resulting from SbEPF2 expression. However, the reduction of stomata within a file was greater (adaxial –24%, abaxial –19%; Fig. 2C, F) than the reduction in the density of files containing stomata (adaxial –21%, abaxial –9%; Fig. 2A, D) on both leaf surfaces. Additional analyses will be needed to fully understand if the reduced density of cell files containing stomata results from changes in cell identity or cell expansion. Cell expansion seems the most likely candidate mechanism since SHORTROOT acts to regulate the formation of cell files containing stomata (Schuler *et al.*, 2018; Wu *et al.*, 2019), and this occurs upstream of the stomatal development pathway (Zoulias *et al.*, 2018). This notion is also supported by the greater width of intercostal regions that contain stomata without changing the number of stomata-containing files within each region in the SbEPF2 plants (Fig. 2A, B, D, E; Supplementary Fig. S3). Meanwhile, the reduced density of stomata within cell files is consistent with the expression of the SbEPF2 triggering the signaling cascade by which the EPF family is well known to determine stomatal fate in various species (Zoulias *et al.*, 2018).

There is evidence for the interplay of stomatal development with hair cells and other specialized epidermal cells (Torii, 2021; Nunes *et al.*, 2023). However, this broad topic, including specifically whether EPF family members play a role in influencing prickle and bulliform development in grasses, is underexplored. Ubiquitous expression of SbEPF2 in sugarcane did not alter bulliform cell density and patterning (Supplementary Fig. S4), the density of prickle-containing files, or the width of costal regions containing prickles (Fig. 3C, G). Previous studies have reported negative correlations between the number of stomata and the number of hairs or trichomes in *Brachypodium* (Raissig *et al.*, 2016; Abrash *et al.*, 2018), maize (Kong *et al.*, 2021), and tomato (Galdon-Armero *et al.*, 2018). These data suggest that the two classes of cells share common pathways early in their development before a subsequent divergence. Prickle cell density and the density of prickles along a cell file decreased in SbEPF2 plants on both leaf surfaces (Fig. 3). This shared response of stomata and prickles to SbEPF2 expression is consistent with evidence from a YODA mutant in *Brachypodium* with greater stomatal and hair cell density implying that common pathways can be involved in regulating spacing between the two cell types (Abrash *et al.*, 2018). In addition, a hair cell-specific peroxidase regulates the size of both hair cells and stomata in *Brachypodium*, with consequences for *g*_sw_ (Nunes *et al.*, 2023). These studies shed further light on how epidermal patterning is regulated and how changes in the density and size of hairs may alter leaf boundary layer conductance (Schreuder *et al.*, 2001; Roth-Nebelsick *et al.*, 2009) and interact with stomatal anatomy to determine leaf gas exchange fluxes.

Model predictions of *g*_smax_ from anatomical measurements of SD and stomatal complex size for plants expressing SbEPF2 were 25% lower on the adaxial surface and 30% lower on the abaxial surface when compared with the wild type (Fig. 6D, H). This model reinforces the expectation that plants expressing SbEPF2 would have reduced *g*_sw_, as observed in previous studies where EPF expression was modified to reduce SD (Dow *et al.*, 2014; Hepworth *et al.*, 2015; Liu *et al.*, 2015; Shen *et al.*, 2015; Wang *et al.*, 2016; Hughes *et al.*, 2017; Caine *et al.*, 2019; Dunn *et al.*, 2019; Mohammed *et al.*, 2019; Li *et al.*, 2021; Clemens *et al.*, 2022; Jiao *et al.*, 2022; Karavolias *et al.*, 2023). However, both transgenic lines expressing SbEPF2 showed no change in *g*_sw_ compared with the wild-type control (Fig. 5B). This was surprising because meaningful reductions in *g*_sw_ have been observed in other species when SD was reduced to a similar degree to that in sugarcane (Fig. 7), but not unprecedented because one event of an *epfl10* knockout in rice had 20% lower SD but showed no change in *g*_sw_ with similar-sized stomatal complexes and increased pore aperture (Karavolias *et al.*, 2023).

In several previous studies, reductions in SD have been accompanied by increases in stomatal complex size and/or stomatal aperture that were thought to ameliorate the reduction in *g*_sw_ (Büssis *et al*., 2006; Franks *et al.*, 2015; Zhu *et al.*, 2015; Wang *et al.*, 2016; Caine *et al.*, 2019; Mohammed *et al.*, 2019; Zhao *et al.*, 2020; Li *et al.*, 2021; Karavolias *et al.*, 2023). However, other studies have reported no such compensating changes in stomatal anatomy, stomatal physiology, or reductions in stomatal complex size that would tend to increase reductions in *g*_sw_ (Hughes *et al.* 2017; Caine *et al.* 2019). Notably, greater stomatal apertures could result from two different but not mutually exclusive mechanisms. First, if larger stomatal complexes develop on low-SD plants and open to the same fraction of their maximum aperture, their aperture would be greater than that of the wild type. Second, guard cells act to open stomata to a larger fraction of their maximum aperture than the wild type without any change in stomatal complex size. The mechanisms underpinning these two responses in plants engineered to have lower SD are unknown. Addressing this knowledge gap is potentially challenging because the contribution of the two mechanisms seems to vary between species and even genotypes or experiments. This variation in *g*_sw_ is the result of complex interactions between SD, stomatal complex size, and stomatal opening (Franks *et al.*, 2009), all of which are dynamically influenced by environmental conditions and *A*_n_ (Long and Bernacchi, 2003; Wolz *et al.*, 2017).

The sugarcane expressing SbEPF2 developed in this present study is a potentially valuable system to study further how stomatal behavior changes in plants engineered to have low SD. First, the absence of any change in *g*_sw_ despite 38% and 26% reductions in SD on the adaxial and abaxial surfaces, respectively, means that there would be no change in transpiration and no feedback effects on water relations that would result from changes in the rate of plant water use. Second, in a C_4_ system under contemporary atmospheric [CO_2_], if modest reductions in *g*_sw_ did occur, they would not increase stomatal limitation to *A*_n_, and feedback effects from altered carbon gain would not occur (Leakey *et al.*, 2019). Third, SbEPF2-expressing plants did not differ from the wild type in proxies for photosynthetic capacity (Fig. 5), which can influence *g*_sw_ in other systems (Franks *et al.*, 2015; Ferguson *et al.*, 2024). Under these circumstances, there was no change in the size of the stomatal complex in sugarcane expressing SbEPF2 (Fig. 4). The most parsimonious explanation for no response in operating *g*_sw_ to the reduction in SD is an increase in stomatal aperture. Stomatal aperture has been estimated either by pressing imprint material onto leaf surfaces in a way that captures a mold of the stomatal pore that can be measured under a microscope (Büssis *et al.*, 2006) or by using a razor blade to dissect away leaf tissue, leaving an epidermal peel where the pore aperture can be assessed directly under a microscope (Karavolias *et al.*, 2023). Unfortunately, these techniques were ineffective in sugarcane because the pore apertures were too small to be reliably visible in imprints. These issues indicate that new methods are needed for assessing pore apertures across wide ranges of species and environments. In the meantime, measurements of leaf gas exchange at very low [CO_2_] and saturating PPFD were used to assess *g*_sw_ when the stomatal aperture is close to its operational maximum. Under those conditions operating *g*_sw_ was 24–28% lower in SbEPF2-expressing lines than in the wild type (Fig. 6). This closely corresponds to the 24% and 30% reductions in *g*_smax_ predicted for the adaxial and abaxial surfaces, respectively, from stomatal anatomy (Fig. 4). This contrasts strongly with *g*_sw_ at ambient [CO_2_] where there was no difference in *g*_sw_ between low-SD and wild-type plants. Stomatal aperture is the only component of *g*_sw_ that can vary across the measurements of [CO_2_]. Therefore, we conclude that a change in stomatal behavior is entirely responsible for fully compensating for the effect of reduced SD on *g*_sw_.

At this time, when parallel studies engineering low SD through manipulation of EPFs and related genes have been done in ≥10 species, there is an opportunity to explore general principles through meta-analysis. We found that across three major plant functional types (C_3_ dicots, C_3_ monocots, and C_4_ monocots), many studies observed that engineered reductions in SD did not reduce *g*_sw_ to an equivalent magnitude (Fig. 7). This suggests that the compensatory effects of larger stomatal complexes and/or altered stomatal aperture are probably more widespread than the previous experimental data on this trait suggest. Although this was especially true in the two monocot groups, the greater variability among experiments on C_3_ dicots means that further work is needed, ideally with multiple species grown side-by-side, to assess if differences among the functional groups are significant and consequential. In C_4_ species, the *x*-axis intercept of the regression relationship suggests that SD must be reduced by >25% to start reducing *g*_sw_. Beyond that threshold, for every 10% reduction in SD, there was a 12% reduction in *g*_sw_. These data provide insight into the reduction of SD required to achieve a targeted reduction in *g*_sw_. Fully understanding the relationship between SD and *g*_sw_ is especially significant when considering that increasingly substantial reductions in *g*_s_ will be desirable to maximize gains in iWUE of all functional types as atmospheric [CO_2_] continues to rise (Leakey *et al.*, 2019).

In conclusion, this report assessed the relationship between SD and *g*_sw_ in sugarcane engineered to have reduced SD. Although improvements in iWUE were not achieved, the study highlighted compensatory mechanisms that operate widely across species and functional groups to diminish the degree to which reductions in SD translate into reductions in *g*_sw_. These findings are important since they highlight a fundamental knowledge gap in regulating leaf gas exchange, which can be addressed to advance our efforts to improve crop iWUE.

## Supplementary data

The following supplementary data are available at *JXB* online.

Fig. S1. Screening transgenic SbEPF2-expressing sugarcane events for low stomatal density.

Fig. S2. Expression of wild-type (WT) transgenic sugarcane overexpressing SbEPF2 (SbEPF2-15 and SbEPF2-38).

Fig. S3. Stomatal regions with more than one stomatal-containing file in wild-type (WT) transgenic sugarcane overexpressing SbEPF2 (SbEPF2-15).

Fig. S4. Epidermal patterning of bulliform cells in wild type (WT) and SbEPF2-expressing sugarcane (SbEPF2-15).

Fig. S5. Abaxial prickle regions with more than one prickle-containing file in wild-type (WT) transgenic sugarcane overexpressing SbEPF2 (SbEPF2-15).

erae271_suppl_Supplementary_Figures_S1-S5

## Data Availability

All data supporting the findings of this study are included in the manuscript and supplementary material. Raw data supporting these figures is available through the University of Illinois Urbana-Champaign data bank under the title ‘Greater aperture counteracts effects of reduced stomatal density on water use efficiency: a case study on sugarcane and meta-analysis https://doi.org/10.13012/B2IDB-9701546_V1.

## Abbreviations:

*A*_n_: net photosynthetic CO2 assimilation
*g*_sw_: stomatal conductance
iWUE: intrinsic water use efficiency
SD: stomatal density.
