# Supplementary material for: Greater aperture counteracts effects of reduced stomatal density on water use efficiency: a case study on sugarcane and meta-analysis
Source: J Exp Bot. 2024 Jul 18;75(21):6837–49. doi: 10.1093/jxb/erae271 (PMC11565199; doi:10.1093/jxb/erae271)
Supplement: erae271_suppl_Supplementary_Figures_S1-S5 [file erae271_suppl_supplementary_figures_s1-s5.pdf]

Greater aperture counteracts effects of reduced stomatal density on WUE: a case study on sugarcane and meta-analysis

Daniel Lunn[1,2,3,4], Baskaran Kannan[5,6], Amandine Germon[1,2,‡, ǁ], Alistair Leverett[1,2,†], Tom E. Clemente[7,8], Fredy Altpeter[5,6], Andrew D. B. Leakey[1,2,3,4,9]

1. Carl R. Woese, Institute of Genomic Biology, 1206 W. Gregory Dr., University of Illinois Urbana-Champaign, Urbana, IL 61801, USA.
2. Center for Advanced Bioenergy and Bioproducts Innovation, 1206 W. Gregory Dr., University of Illinois Urbana-Champaign, Urbana, IL 61801, USA.
3. Department of Plant Biology, University of Illinois Urbana-Champaign, Urbana, IL 61801, USA.
4. Center for Digital Agriculture, University of Illinois Urbana-Champaign, Urbana, IL 61801, USA.
5. Agronomy Department, 3105 McCarty Hall B, University of Florida, Gainesville, FL 32603, USA.
6. Center for Advanced Bioenergy and Bioproducts Innovation, 3105 McCarty Hall B, University of Florida, Gainesville, FL 32603, USA.
7. Department of Agronomy and Horticulture, 202 Keim Hall, University of Nebraska-Lincoln, Lincoln, NE 68583, USA.
8. Center for Advanced Bioenergy and Bioproducts Innovation, 202 Keim Hall, University of Nebraska-Lincoln, Lincoln, NE 68583, USA.
9. Department of Plant Biology, University of Illinois Urbana-Champaign, Urbana, IL, 61801, USA.

[†]Present address: Department of Plant Science, University of Cambridge, Downing Street, Cambridge, CB2 3EA, UK.

[‡]Present address: UMR Eco&Sols, Univ Montpellier, CIRAD, INRAE, IRD, InstitutAgro Montpellier, Montpellier, France.

[ǁ]Present address: CIRAD, UMR Eco&Sols, Montpellier F-34398, France

Author emails: Daniel Lunn (dtl@illinois.edu), Baskaran Kannan (kbaskaran@ufl.edu), Amandine Germon (amandine.germon@cirad.fr), Alistair Leverett (al2178@cam.ac.uk), Tom Clemente (tclemente1@unl.edu), Fredy Altpeter (altpeter@ufl.edu), Andrew D., B., Leakey (leakey@illinois.edu)

Supplementary data:

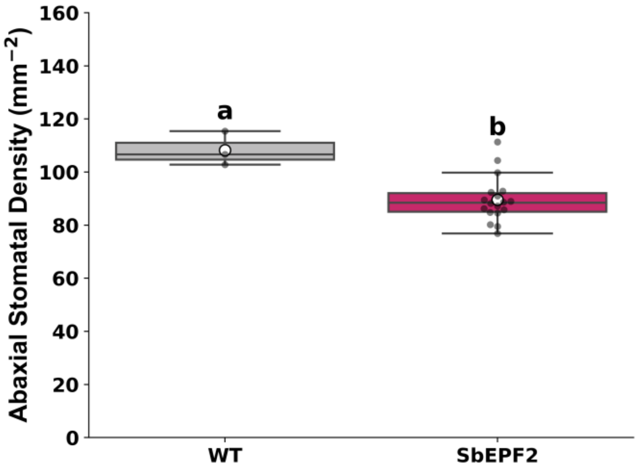

Fig. S1: Screening of wildtype (WT) transgenic sugarcane over-expressing SbEPF2 for low stomatal density. Stomatal density (mm^{-2}) of WT and 18 independent sugarcane transgenic events. Graph show box plots of the 25th and 75th percentile with a line indicating the median value. Each box plot shows the mean with a white circle and grey dots showing individual observed residual data points (n=10) where letters within each panel indicate significant differences between genotypes.

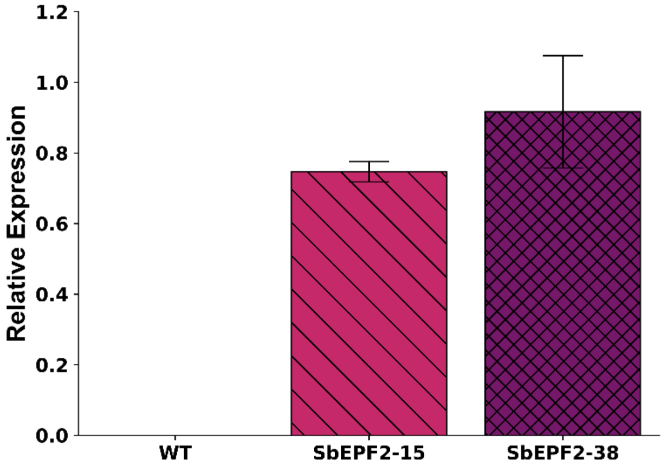

Fig. S2: Expression of wildtype (WT) transgenic sugarcane over-expressing SbEPF2 (SbEPF2-15 and SbEPF2-38). Expression of SbEPF2 in the leaf development zone of three by RT-qPCR using primer set CGACGAGCTAGCAGGAAGAG (forward) and

GGGGATCCTGTGATGTGAGC (reverse). Units are relative expression against the ShGAPDH housekeeping gene using the primer set CACGGCCACTGGAAGCA (forward) and TCCTCAGGGTTCCTGATGCC (reverse). Error bars show standard error with n=3.

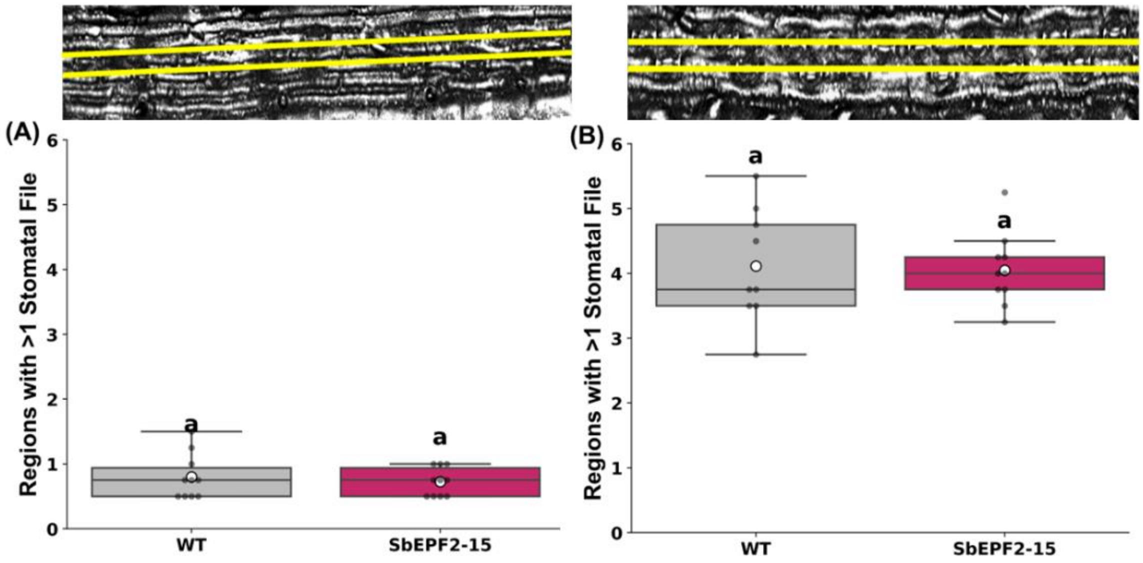

Fig. S3: Stomatal regions with more than one stomatal-containing file in wildtype (WT) transgenic sugarcane over-expressing SbEPF2 (SbEPF2-15). Panels show **(A)** adaxial and **(B)** abaxial stomatal regions with more than one stomatal-containing file. Graphs show box plots of the 25th and 75th percentile with a line indicating the median value. Each box plot shows the mean with a white circle and grey dots showing individual observed residual data points (n=10) where letters within each panel indicate significant differences between genotypes.

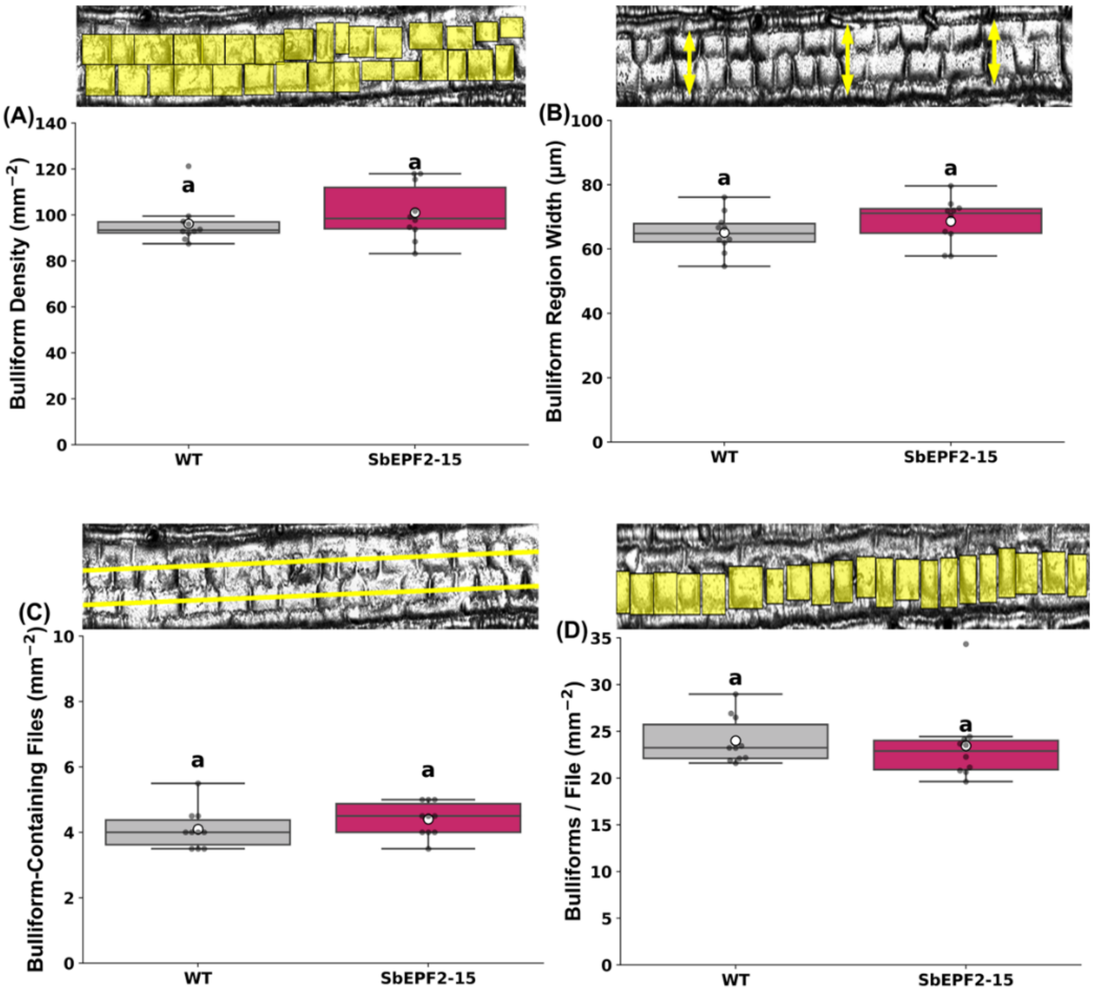

Fig. S4: Bulliform epidermal patterning of wildtype (WT) transgenic sugarcane over-expressing SbEPF2 (SbEPF2-15). Panels show **(A)** bulliform density (mm^2), **(B)** bulliform region width (µm), **(C)** bulliform-containing files, and **(D)** bulliform cells per file. Images above each panel show an example of the schema used to score each trait. Graphs show box plots of the 25th and 75th percentile with a line indicating the median value. Each box plot shows the mean with a white circle and grey dots showing individual observed residual data points (n=10) where letters within each panel indicate significant differences between genotypes.

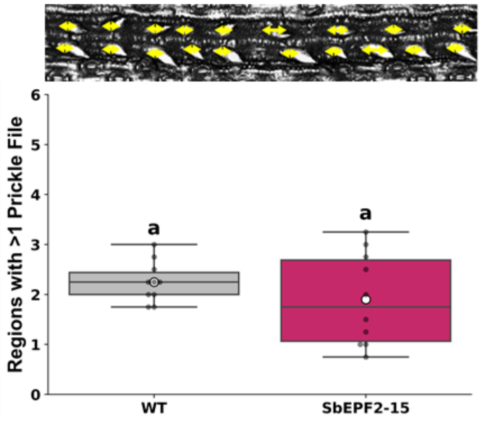

Fig. S5: Abaxial prickle regions with more than one prickle-containing file in wildtype (WT) transgenic sugarcane over-expressing SbEPF2 (SbEPF2-15). Graphs show box plots of the 25th and 75th percentile with a line indicating the median value. Each box plot shows the mean with a white circle and grey dots showing individual observed residual data points (n=10) where letters within each panel indicate significant differences between genotypes.
